# The Pseudotargeted Metabolomics Study on the Toxicity of Fuzi Using Ultraperformance Liquid Chromatography Tandem Mass Spectrometry

**DOI:** 10.1155/2022/6539675

**Published:** 2022-09-13

**Authors:** Huifei Wu, Wenxia Zhang, Hui Lin, Qiuming Ye, Jiayin Guo, Shijian Quan

**Affiliations:** ^1^Zhongshan Hospital of Traditional Chinese Medicine, Zhongshan 528400, China; ^2^Guangdong Provincial Key Laboratory of Drug Screening, School of Pharmaceutical Sciences, Southern Medical University, Guangzhou 510515, China; ^3^Guangzhou University of Chinese Medicine, Guangzhou 510006, China

## Abstract

Fuzi is commonly used in traditional Chinese medicine. Clinical Fuzi poisoning cases have frequently been reported. Glycyrrhizae Radix is often used to alleviate Fuzi's toxicity. However, the poisoning mechanism of Fuzi and the detoxication mechanism of Glycyrrhizae Radix are still not clear. We identified the chemical components of Fuzi at different decoction times (0.5, 1, 2, 4, and 6 h) using ultrahigh performance liquid chromatography quadrupole time-of-flight mass spectrometry. A total of 35 compounds were detected in the Fuzi decoction, including diester alkaloids, monoester alkaloids, amino acids, phenolic acids, organic acids, glycosides, and sugars among others. The content of diester alkaloids (i.e., subaconitine, neoaconitine, and aconitine) in the Fuzi decoction decreased after 2 h of decoction time, while the content of monoester alkaloids (i.e., benzoyl aconitine and benzoyl subaconitine) reached a peak at 2 h. A total of 32 rats were randomly divided into four groups, including 8 cases in the low-dosage Fuzi decoction group A, 8 cases in the high-dosage Fuzi decoction group B, 8 cases in the Fuzi and glycyrrhizae decoction group C, and 8 cases in the control group D. The decoction was administered orally for 7 days. Then, a serum was obtained. The metabolites' changes were analyzed in serum metabolomics using liquid chromatography-tandem mass spectrometry (UPLC-MS/MS). Statistical analysis and pathway analysis were used to assess the effects of glycyrrhizae on the metabolic changes induced by Fuzi. The behavioral and biochemical characteristics indicated that Fuzi exhibited toxic effects on rats and their metabolic profiles changed. However, the metabolic profiles of the glycyrrhizae group became similar to those of the control group. These profiles showed that glycyrrhizae can effectively improve Fuzi poisoning rats. Our study demonstrated that the established pseudotargeted metabolomics is a powerful approach for investigating the mechanisms of herbal toxicity.

## 1. Introduction

Traditional Chinese medicine is generally considered natural and harmless [1–3]. *Aconitum carmichaelii* (called Fuzi in Chinese) has positive pharmacological effects on various diseases such as painful joints, collapse, rheumatic fever, bronchial asthma, syncope, edema, various tumors, and diarrhea [4–6]. Fuzi was first recorded in Shennong's *Materia Medica*. However, its medicinal application on a large scope was limited due to the high toxicity risk and narrow therapeutic range [7]. It is important to establish a standardized chemical method that ensures its safe use. The previous reports demonstrated that aconitine, hypaconitine, and neoaconitine were the main toxic components in Fuzi [8–10]. Therefore, the strategy of reducing toxicity and increasing efficiency was developed to meet the needs of clinical applications. There are a variety of chemical components in Fuzi, so we need to understand how to reduce the content of the toxic ingredients and increase the content of active ingredients.

A clear understanding of the mechanism of Fuzi at the selected decoction time is essential to evaluate its safety. Metabolomics is a comprehensive method for diagnosing diseases, discovering biomarkers, and identifying perturbed pathways [11–14]. Sui et al. investigated the mechanism of aconitine's potential neurotoxicity and nephrotoxicity using a UPLC-Q-TOF-based rat serum and urine metabolomics strategy [8]. Shen et al. evaluated the enhancement of the Glycyrrhizae Radix for the hepatic metabolism of hypaconitine using rat liver S9 [15]. Sun et al. developed an NMR-based metabolomics study of the effect of Glycyrrhizae Radix on the attenuation of toxicity in rats induced by Fuzi [16]. In another previous study, nontargeted metabolomics was applied, but it has the weakness of low sensitivity and poor reproducibility [17]. Targeted metabolomics has the advantage of high sensitivity and good reproducibility. However, it has the weakness of low coverage. Therefore, pseudotargeted metabolomics is a promising tool for the high throughput elucidation of metabolic phenotypes [18].

First, nontargeted serum metabolites were used in full scan and data-dependent modes to generate as high of coverage serum metabolite profiling as possible. Secondly, serum metabolite ion pairs were constructed based on the characteristic fragmental ions and corresponding parent ions of the serum metabolites, which were monitored in an MRM mode in the pseudotargeted metabolomics method. This method combines the advantages of nontargeted and targeted metabolomic approaches.

In a previous study, Luo et al. displayed the dynamic variation patterns of the Aconitum alkaloids in Fuzi during the decoction process [7]. However, there have been few comprehensive identifications of Fuzi components at different decoction times, as well as high sensitivity evaluation methods of the toxic mechanism and detoxification mechanism of Glycyrrhizae Radix.

In our study, we explored the relationship between the chemical components of Fuzi with the decoction time to identify the appropriate decoction time using ultrahigh performance liquid chromatography quadrupole time-of-flight mass spectrometry (UPLC-QTOF). Pseudotargeted rat serum metabolomics was used to evaluate the toxic mechanism of Fuzi and the detoxification mechanism of Glycyrrhizae Radix. This study was the first time where Fuzi poisoning was systematically interpreted, thus providing some information for the future clinical use of Fuzi.

## 2. Materials and Methods

### 2.1. Chemicals

HPLC-grade acetonitrile (purity: 99.9%) was supplied by Fisher Scientific (Fair Lawn, NJ, USA). Water was obtained from Watsons. HPLC-grade formic acid (purity: 98%), chloramphenicol (purity: 98%), and clenbuterol (purity: 98%) were purchased from Sigma-Aldrich (St. Louis, MO, USA). Fuzi and Glycyrrhizae Radix were obtained from the Hospital of Traditional Chinese Medicine of Zhongshan (Guangdong, China) and authenticated by Director Wu from the same hospital.

### 2.2. Preparation of the Decoctions

Fuzi (60 g) was immersed in 200 mL water for 30 min and was then boiled for 0.5, 1, 2, 4, and 6 h, respectively. The supernatant was collected by filtration and centrifuged for 15 min at 4000 g. It was then concentrated to a final volume of 20 mL. The preparation of the Glycyrrhizae Radix decoction was the same as that for Fuzi. Glycyrrhizae decoction containing 3 g raw material per mL was obtained.

### 2.3. Animal Experiment

Thirty-two male Wistar rats (180–220 g) were supplied by the Southern Medical University Laboratory Animal Center and were allowed to acclimatize in cages for 1 week before the experiment. The rats were randomly divided into four groups (*n* = 8/group) as follows: oral gavage with Fuzi at a dose of 30 g/kg (group A), oral gavage with Fuzi at a dose of 60 g/kg (group B), oral gavage with Fuzi and glycyrrhizae at a dose of 60 g/kg (group C), and oral gavage of the same volume of water to healthy controls (group D) as the other three groups. All the groups were given intragastric administrations twice a day for 7 days.

### 2.4. Collection and Preparation of Serum Samples

Serum samples were collected from the retro-orbital venous plexus 7 days after administration. Then, 300 *μ*L acetonitrile was added into 100 *μ*L serum and vortex-mixed for about 5 min for protein precipitation. The mixture was centrifuged at 14000 g for 15 min. Finally, 5 *μ*L aliquots of the supernatant were used for analysis.

### 2.5. Chromatography and Mass Spectrometry

#### 2.5.1. Fuzi Decoction Analysis

Chromatography separation of Fuzi decoction was performed on a Waters T3 column (2.1 mm ^*∗*^ 100 mm, 1.8 *μ*m) using a SCIEX ExionLC AD UPLC system (CA, USA). The column temperature was maintained at 40°C, and the mobile phase consisted of 0.1% formic acid in water (Phase A) and acetonitrile (Phase B) at a constant flow rate of 0.3 mL/min. The injection volume was 5 *μ*L. The 45 min binary gradient elution conditions were optimized as follows: linear gradient from 5% to 10% B (0.5–5 min), 10% to 95% B (5–35 min), 95% to 5% B (40-40.1 min), and then the column was returned to its starting conditions of 5% B for 5 min to allow for column re-equilibration. SCIEX X500R QTOF mass spectrometry (CA, USA) was used to analyze the components of the Fuzi decoction. The optimized MS conditions were as follows: TOF MS scan range: *m/z* 70–1500; TOF MS/MS scan range: *m/z* 50–1500; curtain gas: 35 psi; nebulizer gas: 55 psi; heater gas: 50 psi; temperature: 550°C; ion spray voltage: 5500 V (positive mode)/−4500 V (negative mode); declustering potential: 100 V (positive mode)/−100 V (negative mode); collision potential: 40 ± 20 eV (positive mode)/−40 ± 20 eV (negative mode). A typical information-dependent acquisition (IDA) process was used to carry out the MS/MS experiment.

#### 2.5.2. Pseudotargeted Metabolomics Analysis

Chromatography separation of serum samples was performed on a Waters C18 BEH column (2.1 mm ^*∗*^ 100 mm, 1.7 *μ*m) using a SCIEX ExionLC AD UPLC system (CA, USA). The column temperature was maintained at 40°C, and the mobile phase consisted of 0.1% formic acid in water (Phase A) and acetonitrile (Phase B) at a constant flow rate of 0.4 mL/min. The injection volume was 10 *μ*L. The 11 min binary gradient elution conditions were optimized as follows: linear gradient from 5% to 95% B (1–7 min), 95% to 5% B (9.5-9.6 min), and then the column was returned to its starting conditions of 5% B for 1.5 min to allow for column re-equilibration. SCIEX 4000 QTrap mass spectrometry (CA, USA) was used to detect the metabolites of serum samples. The optimized MS conditions were as follows: curtain gas: 35 psi; nebulizer gas: 55 psi; heater gas: 50 psi; temperature: 550°C; ion spray voltage: 5500 V (positive mode)/−4500 V (negative mode). The MRM transitions of 166 metabolites are shown in the Supplementary Materials (MRMs), which include the metabolite name, their parent ions (Q1), and product ions (Q3).

### 2.6. Data Processing and Statistical Analysis

The components of Fuzi were identified by searching the SCIEX commercialization database using SCIEX OS software. The serum samples were analyzed using MultiQuant 3.0.3 software (CA, USA). Multivariate statistical analysis was performed on MetaboAnalyst 4.0 (Xia Lab at McGill University, Montreal, QC, Canada). Partial least-squares discriminant analysis (PLS-DA) was used to model all features of the four groups. All the metabolites with a significance threshold that satisfies the corrected *p* value cut-off of 0.05 in one-way ANOVA were considered as potential biomarkers.

## 3. Results

### 3.1. Identification of Fuzi Components

**Figure 1:**
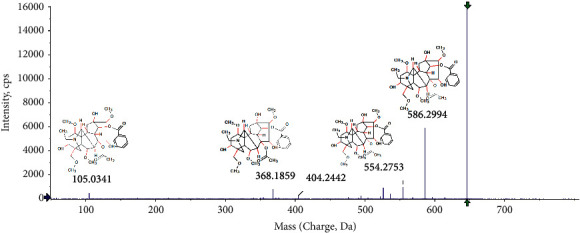
The fragmentation pattern of aconitine.

All components of Fuzi were represented as chromatographic peaks. The parent ions and their product ions were obtained for structural identification. To illustrate the identification of components, we took the *m/z* 646.3233 (*t*_*R*_ = 20.72 min) as an example to be described as follows. Its molecular formula was speculated to be C_34_H_47_NO_11_ based on its parent ion and isotope abundance. In the positive mode, its product ions were observed at *m/z* 586.2994, 554.2753, 368.1859, and 105.0341, which could be obtained by neutral loss of –C_2_H_4_O_2_, –C_3_H_8_O_3_, –C_14_H_32_O_5,_ and –C_27_H_43_NO_10_, respectively. Finally, according to the SCIEX Commercialize Traditional Chinese Medicine database, the ion was tentatively identified as aconitine. The fragmentation pattern of aconitine is shown in Figure 1. The components of Fuzi decoction were identified and are listed in Table 1.

### 3.2. The Comparison of Fuzi Components at Different Decoction times

The Fuzi decoction times were 0.5, 1, 2, 4, and 6 h, respectively. The content of the components was compared at different decoction times, as can be seen in Table 2. Hypaconitine, mesaconitine, and aconitine are diester alkaloids with strong toxicity. In our study, their content increased from 0 h to 1 h of decoction time and decreased after 2 h of decoction time. The results showed that diester alkaloids were thermally unstable. The content of benzoylhypacoitine, benzoylmesaconine, hydroxypurine, adenine, adenosine, and ferulic acid increased from 0.5 h to 2 h of decoction time and decreased after 2 h of decoction time. The content of neoandrographolide, p-coumaric acid, trigonelline, higenamine, and tuberostemonine increased from 0.5 to 1 h of decoction time. Phenprobamate, leucine, L(+)-arginine, L-valine, L-tryptophan, caffeic acid, vanillic acid, citric acid, amber acid, succinic acid, L-malic acid, salidroside, salidroside, guanosine, maltopentaose, D-galactose, D-(+)-mannose, nicotinic acid, 6-methyl coumarin, neoandrographolide, methyl 4-hydroxybenzoate, and norcantharidin can all be detected at the decoction time of 2 h. Therefore, the Fuzi decoction at 2 h was used for the metabolomics study.

### 3.3. Multivariate Statistical Analysis

**Figure 2:**
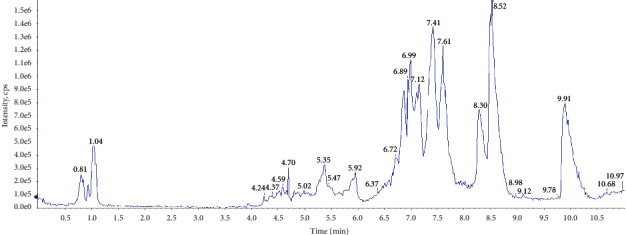
The TIC chromatograms of serum samples.

**Figure 3:**
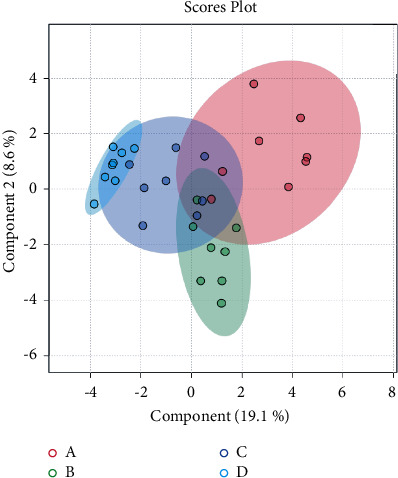
The partial least-squares discriminant analysis recognition method is based on serum metabolomic profiling.

**Figure 4:**
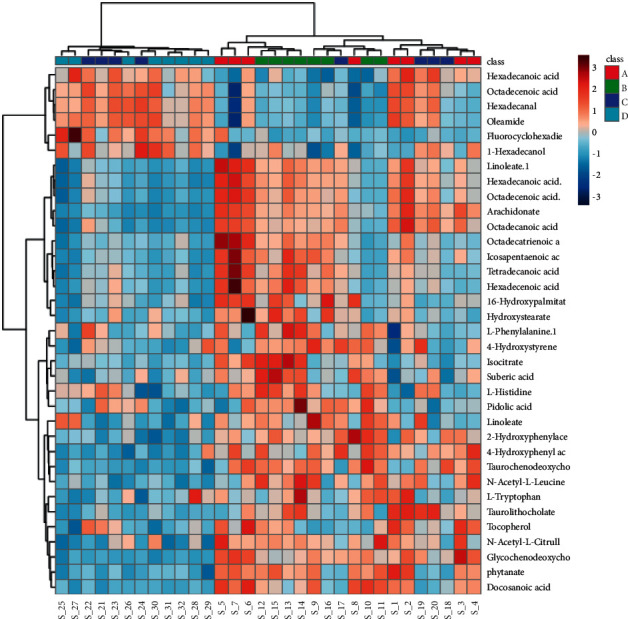
The hierarchical clustering heatmap of the potential biomarkers.

**Figure 5:**
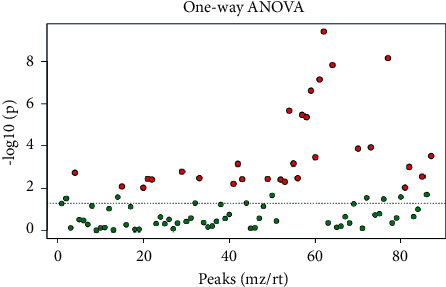
One-way ANOVA of the four groups.

**Figure 6:**
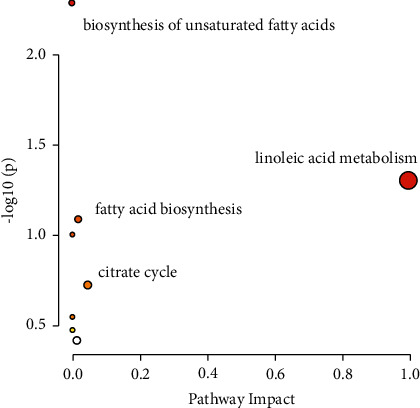
The metabolic pathways related to Fuzi poisoning.

Serum samples were divided into the following four groups: group A (30 g/kg dosage of Fuzi), group B (60 g/kg dosage of Fuzi), group C (60 g/kg dosage of Fuzi and 60 g/kg dosage of glycyrrhizae), and group D (control). The multireaction monitor (MRM) trigger enhanced product ion mode was applied to detect the serum metabolites. Typical serum chromatograms are shown in Figure 2. Chromatographic peaks of metabolites were extracted for alignment and normalization. Then, multivariate statistical analysis was carried out. LPS-DA was applied to model the four groups. As shown in Figure 3, the control group was well separated from groups A and B, suggesting that metabolic perturbation occurred significantly in the Fuzi group. Group C was closer to the control group, suggesting that glycyrrhizae could reduce the Fuzi-induced metabolic perturbation. As shown in Figure 4, the heatmap based on the intensity levels of the metabolites among the four groups was used to clearly characterize the serum metabolites' profile. All differentiated metabolites (*p* < 0.05) in one-way ANOVA were selected. The one-way ANOVA plot is shown in Figure 5. The differential metabolites were hexadecanol, 2-hydroxyphenylacetate, 4-hydroxyphenyl acetate, 16-hydroxypalmitate, docosanoic acid, hexadecanal, hexadecanoic acid, hexadecenoic acid, icosapentaenoic acid, citrate, linoleate, N-acetyl-L-citrulline, N-acetyl-L-leucine, octadecatrienoic acid, octadecenoic acid, oleamide, phytanate, fluorocyclohexadiene, glycochenodeoxycholate-7-sulfate, suberic acid, taurochenodeoxycholate, and tetradecanoic acid. Pathway analysis was used to explore the metabolic pathway related to Fuzi toxicity. Linoleic acid metabolism, biosynthesis of unsaturated fatty acids, fatty acid biosynthesis, and the citrate cycle were disordered after the oral gavage of Fuzi (Figure 6).

## 4. Discussion

A simple, efficient, and sensitive method was established to identify the components of Fuzi decoction at different decocting times using the X500R QTOF system. A total of 35 compounds were found, including 3 diester alkaloids, 2 monoester alkaloids, 3 other alkaloids, 3 base compounds, 6 amino acids, 4 phenolic acids, 3 organic acids, 3 glycosides, and 3 sugars. There were also 5 other categories examined (1 vitamin, 1 coumarin, 1 lactone, 1 ester, and 1 anhydride). The results showed that the diester alkaloids in Fuzi gradually increased and reached the peak in 1∼2 h and then decreased significantly. The monoester alkaloids also gradually increased and reached the peak at 2 h and then decreased significantly. Diester alkaloids with high toxicity are thermally unstable. They can be transformed into monoester alkaloids and further transformed into other alkaloids. This suggests that Fuzi can effectively reduce toxicity after a decocting time of more than 2 h, thus providing useful information for the clinical use of Fuzi.

When used in clinical settings, Fuzi could cause cardiotoxins, neurotoxins, nausea, palpitations, dizziness, vomiting, arrhythmia, hypotension, asystole, shock, coma, and neuron apoptosis among others [19, 20]. Aconitine, mesaconitine, and hypaconitine are the pharmacological and toxic components in Fuzi [21, 22]. In order to explore the effect of Fuzi on serum metabolites, the Fuzi decoction at 2 h was used for the metabolomics study. In clinical settings, glycyrrhizae could alleviate the side effects of Fuzi. In this study, it was used to validate the potential biomarker related to the toxicity of Fuzi.

In our study, pseudotargeted metabolomics was used to investigate the effect of glycyrrhizae on Fuzi-induced toxicity. The pseudotargeted method combines nontargeted and targeted analysis, which has proven to be a high-quality and information-rich method [23]. From analyzing the metabolomics study, the levels of 22 differential serum metabolites became abnormal (as seen in the Supplementary Materials section (available here), where there is a box plot chart of 22 metabolites), and the metabolite profiles of 22 candidate biomarkers were obtained from the quantitative analysis of the subjects. The figure was obtained using GraphPad Prism, and the names of the metabolites are shown in the box plot. The box plot consists of the median (i.e., horizontal line) and the interquartile range, and the whiskers indicate the minimum and maximum values unless there are outliers, in which case the whiskers extend to a maximum of 1.5 times the interquartile range. The 17 serum metabolites, including tetradecanoic acid, taurochenodeoxycholate, suberic acid, phytanate, octadecatrienoic acid, N-acetyl-L-leucine, N-acetyl-L-citrulline, linoleate, icosapentaenoic acid, hexadecenoic acid, hexadecenoic acid, glycochenodeoxycholate-7-sulfate, docosanoic acid, isocitrate, 16-hydroxypalmitate, 4-hydroxyphenyl acetate, and 2-hydroxyphenylacetate, were upregulated in groups A and B. Their contents were significantly higher than that of group D. However, their content in group C was closer to that of group D. This indicated that they were all close to the normal level after glycyrrhizae intervention. The 5 serum metabolites, including oleamide, octadecenoic acid, hexadecanal, fluorocyclohexadiene, and 1-hexadecanol, were downregulated in groups A and B. Their contents were significantly lower than that of group D. However, the content in group C was closer to that of group D. It also indicated that they were all close to the normal level after glycyrrhizae intervention. Therefore, the 22 differential metabolites were related to Fuzi. When Fuzi treatment was combined with the administration of glycyrrhizae, the concentrations of the 22 differential metabolites returned close to their normal levels. In previous studies, glycyrrhizae could delay the absorption of Fuzi or accelerate the metabolism of aconitine, mesaconitine, and hypaconitine to reduce the toxicity of Fuzi [24–28]. Other mechanisms may also be involved [29]. Sun et al. investigated the effect of glycyrrhizae in the attenuation of toxicity in rats induced by Fuzi using the NMR-based metabonomics method [30]. In our study, after pathway analysis, many metabolic pathways, including linoleic acid metabolism, biosynthesis of unsaturated fatty acids, fatty acid biosynthesis, and citrate cycle, were seriously impacted by Fuzi. Glycyrrhizae could regulate the disrupted citrate cycle (i.e., the central metabolic energy pathway) and fatty acid metabolism.

## 5. Conclusions

In our study, a total of 35 components in Fuzi were identified using UPLC-QTOF. Pseudotargeted metabolomics was used to detect the effects of glycyrrhizae on Fuzi-induced toxicity in rats. The results showed that amino acids and organic acids were significantly altered by Fuzi administration in rats. Glycyrrhizae could mitigate these metabolic changes, indicating that glycyrrhizae administration could reduce the toxicity of Fuzi at the metabolic level. The toxicity of Fuzi could be reduced at more than 2 h of decoction time. Our results demonstrate that glycyrrhizae reduces toxicity at the metabolic level through a series of pathways, such as linoleic acid metabolism, biosynthesis of unsaturated fatty acids, fatty acid biosynthesis, and citrate cycle.

## Figures and Tables

**Table 1:** The identified components of Fuzi.

N.O.	RT (min)	Components	Formula	Ionization	Parent ion (*m/z*)	Mass error (ppm)	Product ions (*m/z*)
1	0.87	L (+)-arginine	C_6_H_14_N_4_O_2_	[M + H]^+^	175.1185	−2.8	130.0981; 116.0708; 70.0645; 60.0558
2	0.90	D-galactose	C_6_H_12_O_6_	[M − H]^−^	179.0565	2.4	89.0309; 71.0209; 59.0188
3	0.90	D-(+)-mannose	C_6_H_12_O_6_	[M − H]^−^	179.0563	0.8	89.0319; 71.0190; 59.0193
4	0.99	Trigonelline	C_7_H_7_NO_2_	[M + H]^+^	138.0549	−0.5	94.0661; 92.0501; 78.0345; 65.0395; 51.0225
5	1.02	L-malic acid	C_4_H_6_O_5_	[M − H]^−^	133.0146	3.0	115.0136; 89.0322; 72.9996; 71.0200
6	1.09	L-valine	C_5_H_11_NO_2_	[M + H]^+^	118.0864	1.5	72.0816; 57.0560; 55.0553
7	1.28	Maltopentaose	C_30_H_52_O_26_	[M − H]^−^	827.2687	1.6	665.2686; 587.2333; 545.2182; 425.1672; 341.1378; 281.1107; 179.0731; 161.0594; 101.0324
8	1.33	Adenine	C_5_H_5_N_5_	[M + H]^+^	136.0614	−2.6	119.0364; 109.0517; 92.0253; 65.0142; 66.0219
9	1.33	Citric acid	C_6_H_8_O_7_	[M − H]^−^	191.0196	−0.7	129.0300; 111.0173; 87.0155; 85.0365; 67.0242; 57.0394
10	1.35	Nicotinic acid	C_6_H_5_NO_2_	[M + H]^+^	124.0397	2.9	80.0514; 78.0343; 53.0395; 51.0219
11	1.59	6-hydroxypurine	C_5_H_4_N_4_O	[M + H]^+^	137.0457	−0.6	119.0362; 110.0358; 94.0406; 82.0416; 65.0141; 55.0300
12	1.62	Leucine	C_6_H_13_NO_2_	[M + H]^+^	132.1021	1.3	86.0968; 72.9396; 56.0489
13	1.65	Amber acid	C_4_H_6_O_4_	[M − H]^−^	117.0194	0.3	99.9335; 73.0359; 55.0237
14	1.66	Isoleucine	C_6_H_13_NO_2_	[M + H]^+^	132.1022	2.1	86.0967; 69.0726
15	2.32	Adenosine	C_10_H_13_N_5_O_4_	[M + H]^+^	268.1033	−2.9	136.0619; 119.0361
16	2.38	Guanosine	C_10_H_13_N_5_O_5_	[M − H]^−^	282.0844	−0.1	150.0548; 133.0265; 108.0301; 80.0314
17	2.97	Phenprobamate	C_9_H_11_NO_2_	[M + H]^+^	166.0863	0.0	120.0809; 103.0544; 91.0548; 77.0388; 51.0231
18	3.78	Tuberostemonine	C_22_H_33_NO_4_	[M + H]^+^	376.2479	−0.9	300.1972; 136.0601
19	5.84	L-tryptophan	C_11_H_12_N_2_O_2_	[M − H]^−^	203.0827	0.6	142.0802; 116.0611; 74.0317
20	7.52	Salidroside	C_14_H_20_O_7_	[M − H]^−^	299.1144	2.7	119.0606; 89.0309; 71.0222; 59.0188
21	7.62	Salidroside + NH3	C_14_H_20_O_7_·NH_3_	[M + H]^+^	318.1555	2.4	205.0856; 187.0767; 145.0519; 127.0390; 121.0655; 85.0292
22	7.93	Methyl 4-hydroxybenzoate	C_8_H_8_O_3_	[M − H]^−^	151.0407	3.9	136.0393; 92.0335
23	8.00	p-coumaric acid	C_9_H_8_O_3_	[M − H]^−^	163.0400	−0.3	119.0599; 93.0419
24	8.15	Vanillic acid	C_8_H_8_O_4_	[M − H]^−^	167.0352	1.1	152.0238; 108.0306; 91.0261; 65.0075
25	8.17	Norcantharidin	C_8_H_8_O_4_	[M − H]^−^	167.0354	2.2	152.0263; 108.0310; 91.0250
26	8.21	Higenamine	C_16_H_17_NO_3_	[M + H]^+^	272.1283	0.5	255.1037; 237.0896; 194.0716; 161.0605; 115.0536; 107.0498
27	8.41	6-methylcoumarin	C_10_H_8_O_2_	[M + H]^+^	161.0596	−0.9	115.0559; 89.0378; 79.0554; 51.0240
28	9.00	Caffeic acid	C_9_H_8_O_4_	[M − H]^−^	179.0353	2.0	135.0572
29	9.40	Ferulic acid	C_10_H_10_O_4_	[M + H]^+^	195.0657	2.5	145.0283; 134.0369; 117.0342; 89.0389; 78.0470; 63.0236
30	15.63	Benzoylmesaconine	C_31_H_43_NO_10_	[M + H]^+^	590.2950	−1.7	540.2556; 508.2307; 105.0328
31	17.36	Benzoylhypacoitine	C_31_H_43_NO_9_	[M + H]^+^	574.3003	−1.4	542.2727; 510.2492; 105.0335
32	18.58	Neoandrographolide + HCOOH	C_26_H_40_O_8_·HCOOH	[M − H]^−^	525.2705	0.0	479.2983; 161.0609; 101.0329
33	19.28	Mesaconitine	C_33_H_45_NO_11_	[M + H]^+^	632.3059	−1.0	572.2820; 540.2582; 354.1692; 105.0334
34	20.67	Hypaconitine	C_33_H_45_NO_10_	[M + H]^+^	616.3106	−1.6	556.2872; 524.2615; 492.2377; 338.1737; 105.0335
35	20.72	Aconitine	C_34_H_47_NO_11_	[M + H]^+^	646.3233	1.7	586.2994; 554.2753; 368.1859; 105.0341

**Table 2:** The comparison of Fuzi components at different decoction times.

Sample name	0.5 h	1 h	2 h	4 h	6 h
L(+)-arginine	4224486	6052934	6016773	5458557	4017949
D-galactose	171450	220332	248957	240805	186396
D-(+)-mannose	172431	221452	251645	241490	187498
Trigonelline	913625	1381259	1327159	1090388	660688
L-malic acid	6215	191516	91834	75902	1295
L-valine	72167	73913	85500	94464	181698
Maltopentaose	49923	81807	84371	87473	63982
Adenine	938595	1548668	482508	3005214	28002
Citric acid	788565	1697871	1716444	1421501	783274
Nicotinic acid	19520	68645	35373	49658	48757
6-hydroxypurine	1187035	779049	1196645	173327	19006
Leucine	220014	268314	91526	575355	3036
Amber acid	337516	227553	212178	194989	4811
Isoleucine	220014	268314	91526	575355	3036
Adenosine	27481	69440	17260	1404649	925160
Guanosine	2484	2593	3807	322899	144894
Phenprobamate	679550	1005854	775874	1257039	10856
Tuberostemonine	300892	555456	525932	455063	283580
L-tryptophan	92671	161310	133079	163463	40834
Salidroide	41459	67702	64416	53856	32779
Salidroside + NH_3_	88351	145468	137597	111607	75283
Methyl 4-hydroxybenzoate	9170	27237	28549	30591	20074
p-coumaric acid	374263	537298	518972	407214	263705
Vanillic acid	81285	168227	181606	137018	68543
Norcantharidin	81284	168291	181596	137011	68512
Higenamine	83516	105990	107368	91255	58503
6-methylcoumarin	313149	455988	477787	421839	264078
Caffeic acid	209862	315156	316971	269357	183122
Ferulic acid	259704	375697	400387	312247	104415
Benzoylmesaconine	74271918	111058354	115423724	65234980	62682149
Benzoylhypacoitine	11173798	20504076	25449749	20971006	16650982
Neoandrographolide + HCOOH	143464	259580	224233	188300	73635
Mesaconitine	19635989	25672293	16326032	8592867	3459599
Hypaconitine	34141086	42983172	46925126	34294964	22862038
Aconitine	4638692	6134692	4606976	2414212	1031370

## Data Availability

The datasets generated during the present study are available from the corresponding author on reasonable request.

## Supplementary Materials

Supplementary Materials “Supplementary materials_MRMs” file includes the MRM transitions of 166 metabolites, such as their parent ions (Q1) and product ions (Q3). “Supplementary materials_the 22 metabolites” file includes the levels of 22 differential serum metabolites from the quantitative analysis of the subjects.
