# Supplementary material for: The Pseudotargeted Metabolomics Study on the Toxicity of Fuzi Using Ultraperformance Liquid Chromatography Tandem Mass Spectrometry
Source: Evid Based Complement Alternat Med. 2022 Sep 13;2022:6539675. doi: 10.1155/2022/6539675 (PMC9489361; doi:10.1155/2022/6539675)
Supplement: Supplementary Materials — “Supplementary materials_MRMs” file includes the MRM transitions of 166 metabolites, such as their parent ions (Q1) and product ions (Q3). “Supplementary materials_the 22 metabolites” file includes the levels of 22 differential serum metabolites from the quantitative analysis of the subjects. [file 6539675.f1.zip › Supplementary materials_MRMs.pdf]

compound.name	Q1	Q3_1	Q3_2
Phenol	93.0	65	75
Pidolic acid	128.0	82	52
2-Oxohexanoic acid	129.1	69	83
3-Methyl-2-oxopentanoated	129.1	69	83
4-Methyl-2-oxopentanoate	129.1	69	83
L-Isoleucine	130.1	130	58
L-leucine	130.1	130	88
Threonate	135.0	75	59
4-Hydroxybenzoate	137.0	93	65
Salicylate	137.0	93	65
L-Glutamine	145.1	127	109
L-Glutamate	146.0	102	128
3,4-Dihydroxybenzoate	153.0	109	65
L-Phenylalanine	164.1	103	147
Vanillate	167.0	108	69
N-Acetyl-L-leucine	172.1	130	111
Suberic acid	173.1	111	109
L-Citrulline	174.1	131	
Hippurate	178.1	77	134
L-Tyrosine	180.1	119	163
Azelaic acid	187.1	125	97
Citrate	191.0	111	87
Isocitrate	191.0	111	87
Salicyluric acid	194.0	150	93
Sebacic acid	201.1	139	183
L-Tryptophan	203.1	116	142
Indolelactate	204.1	158	130
N-Acetyl-L-phenylalanine	206.1	164	147
Tetradecanoic acid	227.2	227	
(9Z)-Hexadecenoic acid	253.2	253	
Hexadecanoic acid	255.2	255	
Phenylacetylglutamine	263.1	145	127
16-Hydroxypalmitate	271.2	225	271
(6Z,9Z,12Z)-Octadecatrienoic ac	277.2	277	
(9Z,12Z,15Z)-Octadecatrienoic a	277.2	277	
Linoleate	279.2	279	
(11E)-Octadecenoic acid	281.2	281	
(9Z)-Octadecenoic acid	281.2	281	
Octadecanoic acid	283.3	283	
(5Z,8Z,11Z,14Z,17Z)-Icosapenta	301.2	301	257.2
Arachidonate	303.2	303	259.2
Phytanate	311.3	311	134
Icosanoic acid	311.3	311	134
Erucic acid	337.3	337	
Docosanoic acid	339.3	339	
Estrone 3-sulfate	349.1	269	
Tetrahydrocorticosterone	349.2	305	331
(15Z)-Tetracosenoic acid	365.3	265	
Chenodeoxycholate	391.3	247	391
Deoxycholic acid	391.3	391	
3beta-Hydroxypregn-5-en-20-	395.2	97	395
Cholic acid	407.3	407	
alpha-Tocopherol	429.4	163	429

Glycochenodeoxycholate	448.3	74	448
Glycodeoxycholate	448.3	74	386
Glycocholate	464.3	74	402
Taurolithocholate	482.3	482.3	
Taurochenodeoxycholate	498.3	498.3	
Taurodeoxycholate	498.3	498.3	
Taurocholate	514.3	514.3	
Naringin	579.2	427	339.2
Bilirubin	583.3	285.1	213.1
Thyroxine	775.7	126.9	604.8
Phenyllactate	165.1	119	147
N-Hydroxy-L-tryptophan	219.1	144	132
Chorismate	245.0	165	108
Isochorismate	245.0	165	108
12(13)-EpOME	295.2	277	195
9(10)-EpOME	295.2	277	195
5-(3,4-Diacetoxybut-1-ynyl)-2,2	353.0	273	152
Dehydroepiandrosterone sulfate	367.2	97	367
Hyodeoxycholate	391.3	347.3	329.3
Murideoxycholic acid	391.3	347.3	329.3
3alpha,7alpha-Dihydroxy-12-ox	405.3	325.3	387.3
1-O-[2-(Acetylamino)-2-deoxy	418.1	222	266
Estrone glucuronide	465.2	397	97
S-(Hydroxymethyl)mycothiol	497.1	203	225
Glycochenodeoxycholate 7-sulfa	528.3	528	448
(3Z)-Phytochromobilin	583.3	299	227
15,16-Dihydrobiliverdin	583.3	299	227
2'''-N-Acetyl-6'''-deamino-6'''-	657.3	367.2	657.3
4-Cresol	107.1	77	
Indole	116.1	100	
4-Hydroxystyrene	119.1	93	117
Phenylacetaldehyde	119.1	93	117
4-Hydroxyaniline	144.0	58	
2-Hydroxyphenylacetate	151.0	93	
Methyl salicylate	151.0	93	
1-Fluorocyclohexadiene-cis,cis-	165.0	121	
2-Hydroxy-6-oxoocta-2,4,7-trie	167.0	83	69
3-(2,3-Dihydroxyphenyl)propan	181.1	135	163
Homovanillate	181.1	135	163
2-Hydroxy-6-oxo-7-methylocta	183.1	139	
3-Indoleacrylate	186.1	142	116
4-Hydroxyphenyl acetate	187.0	129	
4-Hydroxyphenyl-4-hydroxyber	229.0	116	57
Stearidonic acid	275.2	139	117
(R)-10-Hydroxystearate	299.3	299	
N-Acetyl-L-citrulline	198.1	130	
Cystathionine	241.1	151	115
Indolepyruvate	243.1	131	142
N(alpha)-gamma-L-Glutamylhis	259.1	131	87
L-Isoleucine	132.1	86	69
L-leucine	132.1	86	91
L-Glutamine	147.1	84	130
L-Histidine	156.1	110	83
L-Valine	159.1	113	72

L-Phenylalanine	166.1	120	103
N6-Acetyl-L-lysine	171.1	84	
L-Arginine	175.1	70	60
Indole-3-acetate	176.1	130	103
Hippurate	180.1	105	77
1,7-Dimethylxanthine	181.1	124	96
Theophylline	181.1	124	96
L-Tyrosine	182.1	91	136
L-Lysine	188.1	84	143
Caffeine	195.1	138	110
Indolelactate	206.1	130	118
Indolepyruvate	221.1	130	91
Phenylacetylglutamine	265.1	130	91
Arachidonate	269.2	93	119
(6Z,9Z,12Z)-Octadecatrienoic ac	279.2	95	67
(9Z,12Z,15Z)-Octadecatrienoic a	279.2	95	67
Linoleate	281.2	81	95
(11E)-Octadecenoic acid	283.3	69	121
(9Z)-Octadecenoic acid	283.3	69	121
(4Z,7Z,10Z,13Z,16Z,19Z)-Docosa	329.2	91	131
Chenodeoxycholate	357.3	105	133
Cholic acid	373.3	269	73
Riboflavin	377.1	243	72
L-Palmitoylcarnitine	400.3	85	341.3
alpha-Tocopherol	431.4	165	
Guanidinoacetate	118.1	58	91
N-Methylaniline	130.1	77	103
3-Oxo-3-ureidopropanoate	188.1	118	115
Coniferyl acetate	223.1	181	139
N8-Acetylspermidine	229.2	95	
Hexadecanoic acid	257.2	57	257
Hexadecanal	282.3	83	69
Oleamide	282.3	83	69
(8E,10S)-10-Hydroperoxyoctade	337.2	225	
9,10-Epoxy-18-hydroxystearate	337.2	225	
3alpha,12alpha-Dihydroxy-5bet	355.3	267	73
Deoxycholic acid	357.3	247	
Hyodeoxycholate	357.3	247	
Murideoxycholic acid	357.3	247	
3-Oxo-5beta-cholanate	375.3	271	
3alpha,7alpha-Dihydroxy-12-ox	389.3	356	
3alpha,12alpha-Dihydroxy-7-ox	407.3	335	389
Urea-1-carboxylate	105.0	77	51
3-Guanidinopropanoate	132.1	117	
4-Hydroxyphenylacetonitrile	134.1	77	
Mandelonitrile	134.1	77	
Dihydrocoumarin	149.1	77	103
Pyruvophenone	149.1	77	103
Phenylpropanoate	151.1	105	79
Methyleugenol	179.1	105	79
Methylisoeugenol	179.1	105	79
9-Oxononanoic acid	195.1	95	77
(1E,3E)-4-Hydroxybuta-1,3-dier	203.0	85	57
Didemethylcitalopram	319.1	241	283

S-Adenosyl-4-methylthio-2-ox	421.1	109	313
Deisopropyldeethylatrazine	184.0	166	108
1-Hexadecanol	284.3	57	135
9,10-EOT	334.2	219	203
Colnelenic acid	334.2	219	203
