# Supplementary figures and images for: The Pseudotargeted Metabolomics Study on the Toxicity of Fuzi Using Ultraperformance Liquid Chromatography Tandem Mass Spectrometry
Source: Evid Based Complement Alternat Med. 2022 Sep 13;2022:6539675. doi: 10.1155/2022/6539675 (PMC9489361; doi:10.1155/2022/6539675)

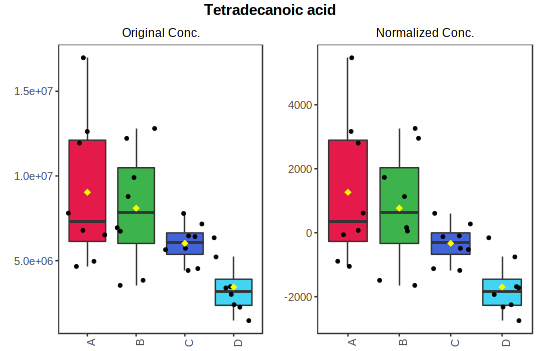

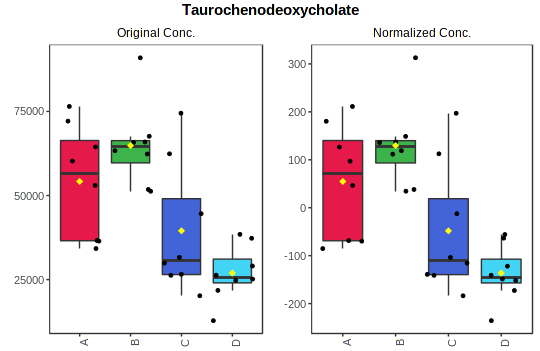

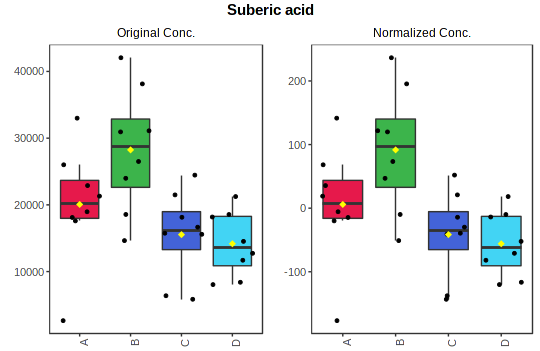

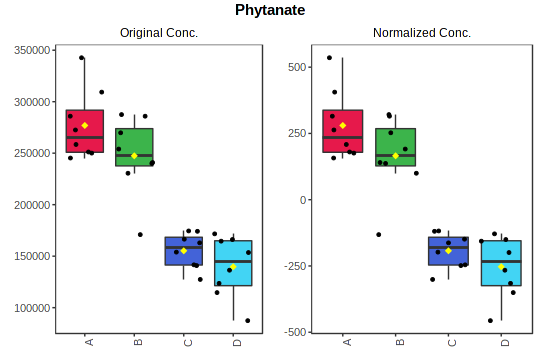

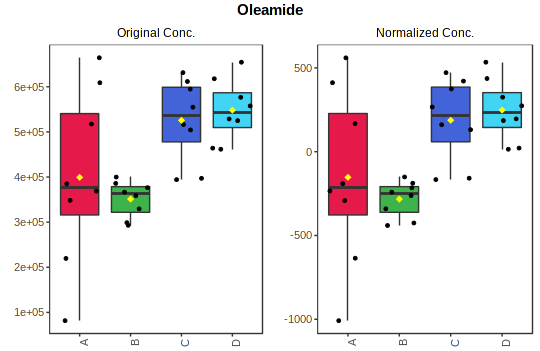

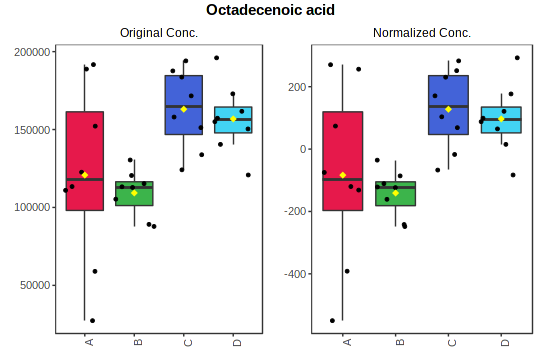

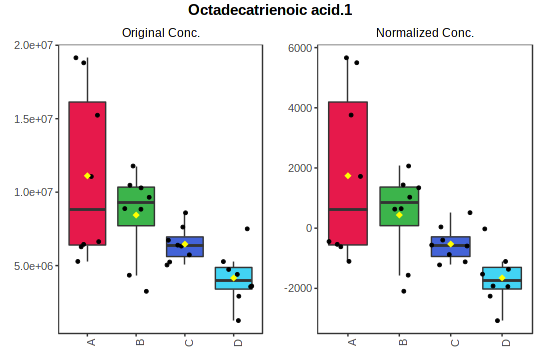

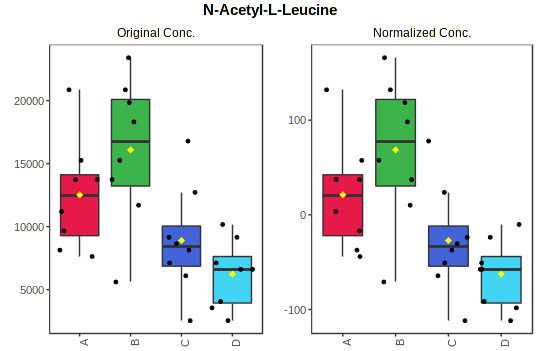

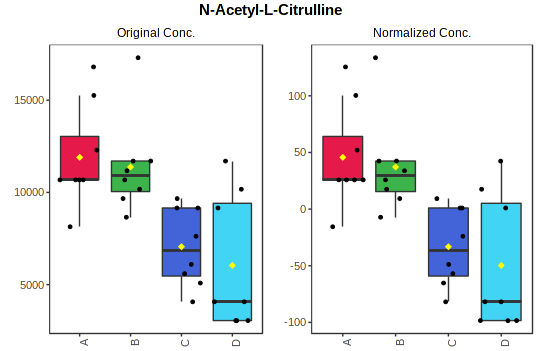

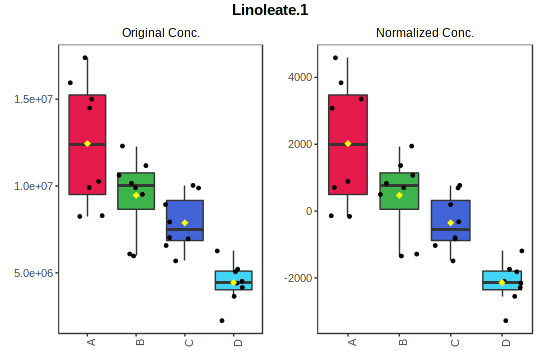

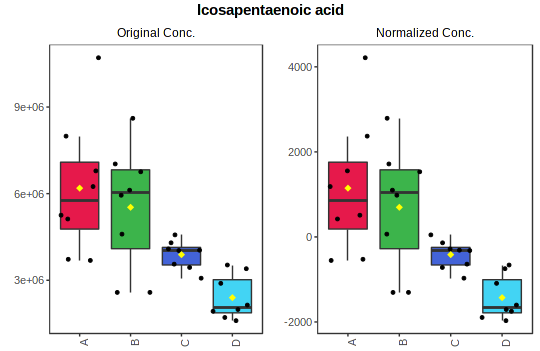

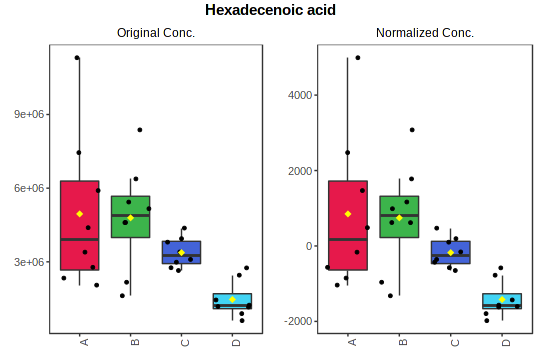

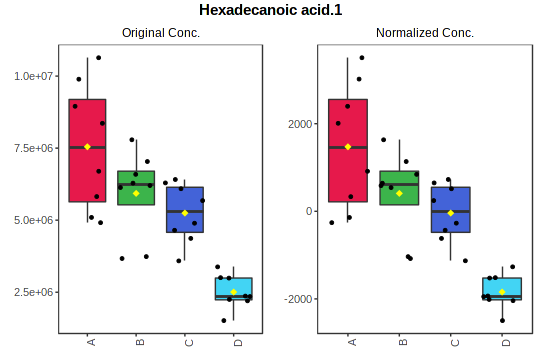

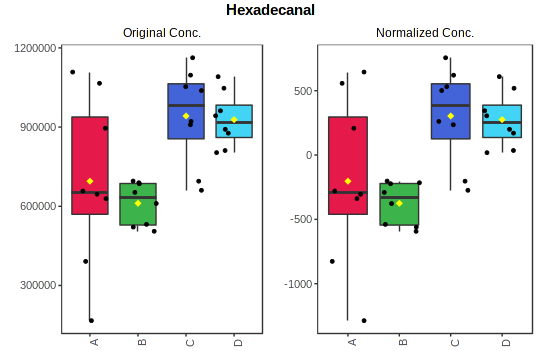

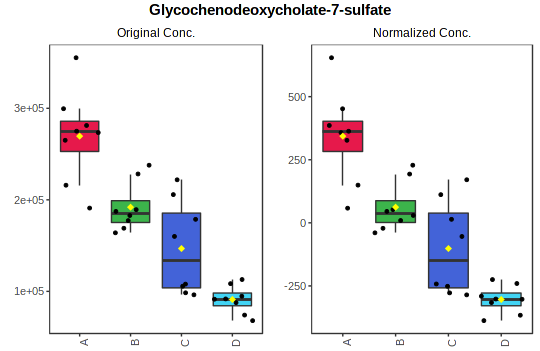

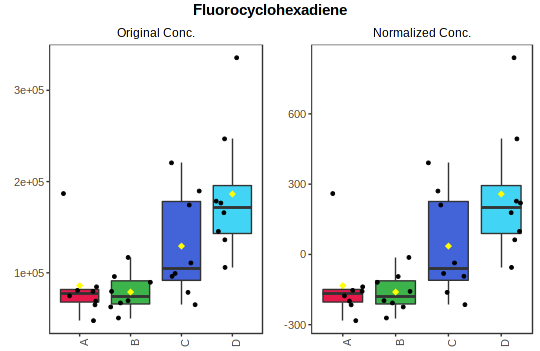

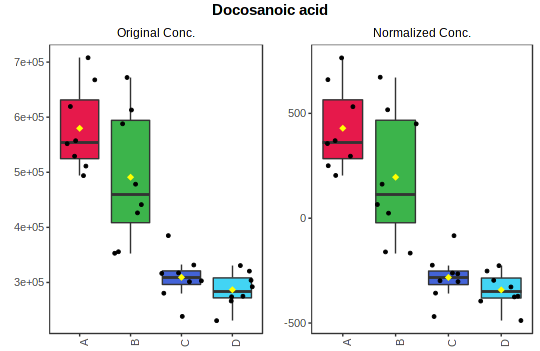

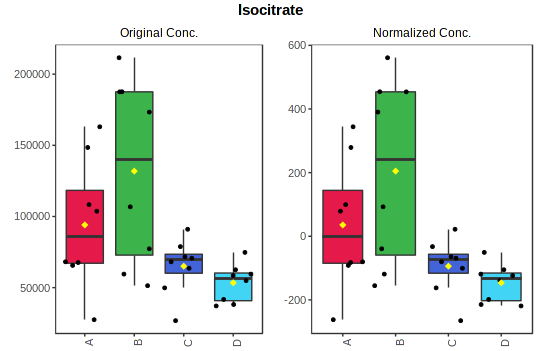

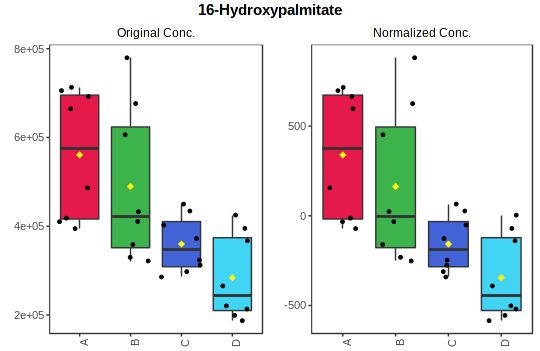

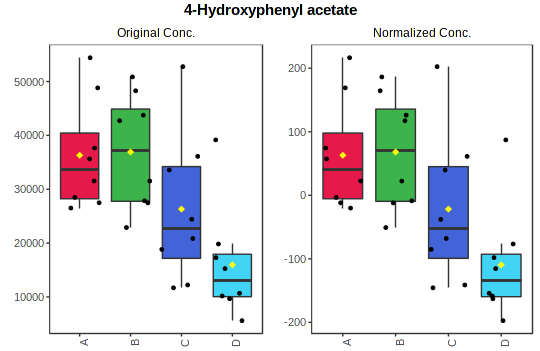

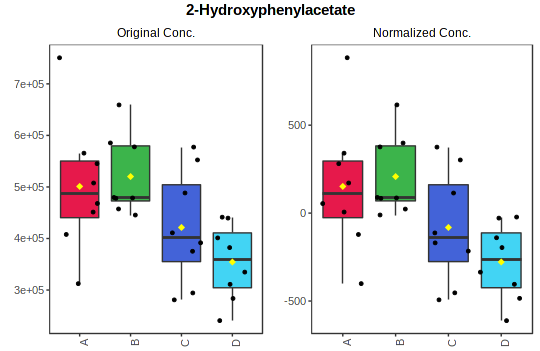

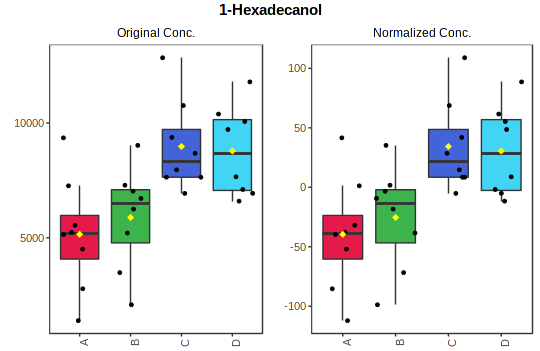

Supplement: Supplementary Materials — “Supplementary materials_MRMs” file includes the MRM transitions of 166 metabolites, such as their parent ions (Q1) and product ions (Q3). “Supplementary materials_the 22 metabolites” file includes the levels of 22 differential serum metabolites from the quantitative analysis of the subjects. [file 6539675.f1.zip › supplementary materials_the 22 metabolites.docx]
